# Low levels of antibodies against common viruses associate with anti-citrullinated protein antibody-positive rheumatoid arthritis; implications for disease aetiology

**DOI:** 10.1186/s13075-017-1423-9

**Published:** 2017-09-30

**Authors:** Natalia Sherina, Hulda S. Hreggvidsdottir, Camilla Bengtsson, Monika Hansson, Lena Israelsson, Lars Alfredsson, Karin Lundberg

**Affiliations:** 1 Department of Medicine, Rheumatology Unit, Karolinska Institutet, Karolinska University Hospital Solna, Stockholm, Sweden; 2 Cardiovascular Epidemiology, Institute of Environmental Medicine, Karolinska Institutet, Stockholm, Sweden; 3 Centre for Occupational and Environmental Medicine, Stockholm County Council, Stockholm, Sweden

**Keywords:** Rheumatoid arthritis (RA), Anti-CCP, Infections, Autoantibodies

## Abstract

**Background:**

Infection by common viruses has long been discussed in the aetiology of a number of autoimmune diseases, including rheumatoid arthritis (RA). However, studies investigating this hypothesis in RA show conflicting results. These studies often lack well-matched control populations, and many do not include data on autoantibodies, genetic risk factors and other environmental factors, which are known to contribute to disease only in subgroups of patients. In the present study, we have therefore examined the role of Epstein-Barr virus (EBV), cytomegalovirus (CMV) and parvovirus B19 (B19) in RA aetiology, by analysing anti-viral antibodies in relation to anti-citrullinated protein antibodies (ACPA), smoking, *HLA-DRB1* shared epitope (SE) alleles, and clinical parameters, in both RA patients and matched controls.

**Methods:**

Anti-viral antibodies were measured by ELISA in serum samples from 990 RA patients and 700 controls from the Swedish population-based Epidemiological Investigation of RA (EIRA) cohort. Data on ACPA, smoking, SE, inflammation (C-reactive protein) and disease activity score in 28 joints (DAS28) was obtained from the EIRA database. Fisher’s exact test, the chi-squared test, and the Mann-Whitney *U* test were used to calculate differences in anti-viral antibody frequencies and levels; unconditional logistic regression was used to determine the association of anti-viral antibodies with different RA subsets.

**Results:**

Antibodies against all viruses were highly prevalent in EIRA, with no major differences detected between ACPA-positive RA, ACPA-negative RA and controls. However, both anti-B19 and anti-EBV IgG levels were significantly lower in ACPA-positive RA compared to controls, and there were significant interactions between low levels of anti-B19 and anti-EBV antibodies and SE in the development of ACPA-positive RA.

**Conclusion:**

We could not detect an association between RA and elevated anti-viral antibody levels, for any of the three common viruses, EBV, CMV or B19. On the contrary, our study demonstrated association between low anti-EBV/anti-B19 antibody levels and ACPA-positive RA, in particular when *HLA-DRB1* SE was present. These data could potentially suggest that high anti-viral antibody levels would be protective against ACPA-positive RA. Further investigations are required to address the mechanisms behind these findings.

**Electronic supplementary material:**

The online version of this article (doi:10.1186/s13075-017-1423-9) contains supplementary material, which is available to authorized users.

## Background

The aetiological association between genes and environmental factors in the development of rheumatoid arthritis (RA) is well-established today, with specific *HLA-DRB1* alleles, termed the shared epitope (SE), as the major genetic contributor [1], and smoking as the main environmental risk factor [2]. Moreover, there is a strong biological interaction between smoking and SE, specifically in anti-citrullinated protein antibody (ACPA)-positive RA [3]. Importantly though, current knowledge does not explain the whole risk of developing RA and there has been an extensive search for additional environmental factors predisposing individuals to RA.

The link between an infectious agent and the development of RA has long been discussed, and three decades ago researchers reported that patients with RA have higher frequency and increased antibody titres against Epstein-Barr virus (EBV) compared to controls [4, 5]. Since then, a number of studies have addressed the possible role of viral infections in the aetiopathogenesis of RA. In addition to EBV [6], other viruses have been implicated, including human parvovirus B19 (B19) [7, 8], and cytomegalovirus (CMV) [9].

EBV and CMV belong to the human herpes virus family. Primary infection is often asymptomatic, but may cause severe morbidity, even mortality, in immunocompromised patients. Infection by parvovirus B19 may cause erythema infectiosum (fifth disease), predominantly affecting children. In adults, B19 often causes self-limiting acute symmetric polyarthritis [10], with symptoms disappearing within a few weeks, although arthralgia may persist for months, even years, in about 20%, usually women [11], suggesting that polyarthritis caused by B19 may progress to RA.

Whereas a number of serological studies have identified higher antibody levels against these viruses and/or increased antibody frequency in RA compared to controls [4, 5, 7, 12], other studies have not replicated these findings [13–15]. In addition to serological evidence, viral DNA has been detected in the synovium and bone marrow of patients with RA [7, 12–23], and studies also report on the presence of viral proteins in the joints of patients with RA [7, 16, 17]. However, other studies have had conflicting results [24–26].

Most studies performed to date, on the association between viral infections and RA development, have used small cohorts and often lack well-matched control groups. With this in mind, it seems difficult to draw any conclusions from the current literature, which presents contradictory results. Furthermore, studies during the past decade have highlighted the importance of autoimmunity to citrullinated proteins, when investigating genetic and environmental risk factors for RA, and such studies have not been conducted when it comes to viral infections.

Using the large population-based Swedish Epidemiological Investigation of Rheumatoid Arthritis (EIRA) case-control study, we had a unique opportunity to analyse anti-viral antibody levels in relation to RA, autoantibodies, genetic risk factors, smoking habits and clinical parameters. Hence, in the present study we have investigated the associations of EBV, B19 and CMV viruses with ACPA-positive and ACPA-negative disease, in relation to SE, smoking status, disease activity score in 28 joints (DAS28) and C-reactive protein (CRP) levels by measuring anti-viral IgG in sera from 990 patients with RA (cases) and 700 matched controls.

## Methods

### Study design

This study is based on 990 patients with RA and 700 age-matched, gender-matched and residential-area-matched controls from the EIRA cohort. Incident cases were diagnosed according to the 1987 American College of Rheumatology criteria [27]. Participants donated blood at inclusion (at the time of diagnosis of RA) and filled in a questionnaire on lifestyle/environment. Patients had not been treated with disease-modifying anti-rheumatic drugs (DMARDs) prior to this time point. Details of EIRA are described elsewhere [2]. Information on ACPA, smoking, genetics and clinical data were retrieved from the EIRA database, and has described before [2, 3, 28, 29]. Briefly, EIRA participants were categorised as ever-smokers (current and former smokers) or never-smokers, and genotyped for *HLA-DRB1* alleles (HLA-DRB1*01 (except DRB1*0103), *04 and *10 were classified as SE). ACPA was measured as anti-CCP2 IgG using Immunoscan CCPlus® (Euro-Diagnostica AB, Malmö, Sweden). Clinical data (DAS28 and CRP) were captured by linking EIRA with the Swedish rheumatology register, where clinical data are registered as part of standard care [29]. The study population is described in Table 1.

**Table 1:** Characteristics of EIRA RA cases and controls

	RA cases (n = 990)	Controls (n = 700)
Age, years, mean (SD)	50.5 (12.7)	51.5 (11.9)
Gender, female/male (% female)	700/290 (71)	500/200 (71)
ACPA positive (%)	601 (61)	N/A
IgM RF positive (%)	653 (66)	N/A
DAS28, median (IQR)	5.07 (4.08-5.91)	N/A
CRP, mg/l, median (IQR)	16.0 (8.0-31.0)	N/A
Smoking status, ever^a^ (%)	558 (56)	355 (51)
*HLA-DRB1* SE positive^b^ (%)	711 (72)	342 (49)

^a^Former and current cigarette smokers.

^b^Carriers of one or two *HLA-DRB1* SE alleles

*ACPA* anti-citrullinated protein antibody, *RF* rheumatoid factor, *CRP* C-reactive protein, *DAS28* disease activity score in 28 joints, *IQR* interquartile range, *N/A* not applicable, *RA* rheumatoid arthritis, *SD* standard deviation, *SE* shared epitope

### Detection of anti-viral IgG in serum

Anti-CMV IgG was analysed using a chemiluminescent microparticle immunoassay (Architect, Abbott Diagnostics, Wiesbaden, Germany). Antibodies against EBV viral capsid antigen, and parvovirus B19, were measured by enzyme-linked immunosorbent assay (ELISA) kits (Biorad, Dreieich, Germany, and Biotrin International, Dublin, Ireland, respectively). All immunoassays were performed and interpreted according to the manufacturers' instructions, including setting of the cutoff values. The cutoff for positivity was ≥ 6.0 arbitrary units (AU)/ml for anti-CMV IgG, and > 1.0 index value for both anti-EBV IgG and anti-B19 IgG. Anti-CMV and anti-EBV antibodies were analysed in 987 patients with RA and 700 controls, while anti-B19 antibodies were analysed in 979 patients with RA and 692 controls.

### Detection of rheumatoid factor in serum

Levels of IgM rheumatoid factor (RF) were analysed in all 990 patients with RA from the EIRA cohort, using the EliA immunoassay on the Phadia 2500 system (Phadia GmbH, Freiburg, Germany), according to the manufacturer's instructions.

### Statistical analysis

Statistical differences in anti-viral antibody frequencies between different RA subsets and controls were determined using Fisher’s exact test and the chi-squared test. The Mann-Whitney *U* test was used to examine anti-viral IgG levels. Linear regression was used to determine the correlation between anti-viral antibody levels and DAS28/CRP, age and RF. Unconditional logistic regression was used to calculate odds ratios (OR) with 95% confidence intervals (CI) for the association between high/low anti-viral antibody levels and different RA subsets. Quartiles of anti-viral antibody levels were used, and the highest quartile (25%) was compared to the lower three quartiles (75%). Cutoffs were based on anti-viral IgG levels among controls. Analyses were adjusted for age, gender and residential area. The attributable proportion (AP) due to additive interaction was evaluated between smoking, SE and virus exposure, regarding the risk of ACPA-positive RA, with 95% CI, as previously described [30]. Analyses were performed using GraphPad PRISM 6 and SAS version 9.3. *P* values <0.05 were considered statistically significant.

## Results

### Prevalence of anti-viral antibodies in EIRA

**Fig. 1:**
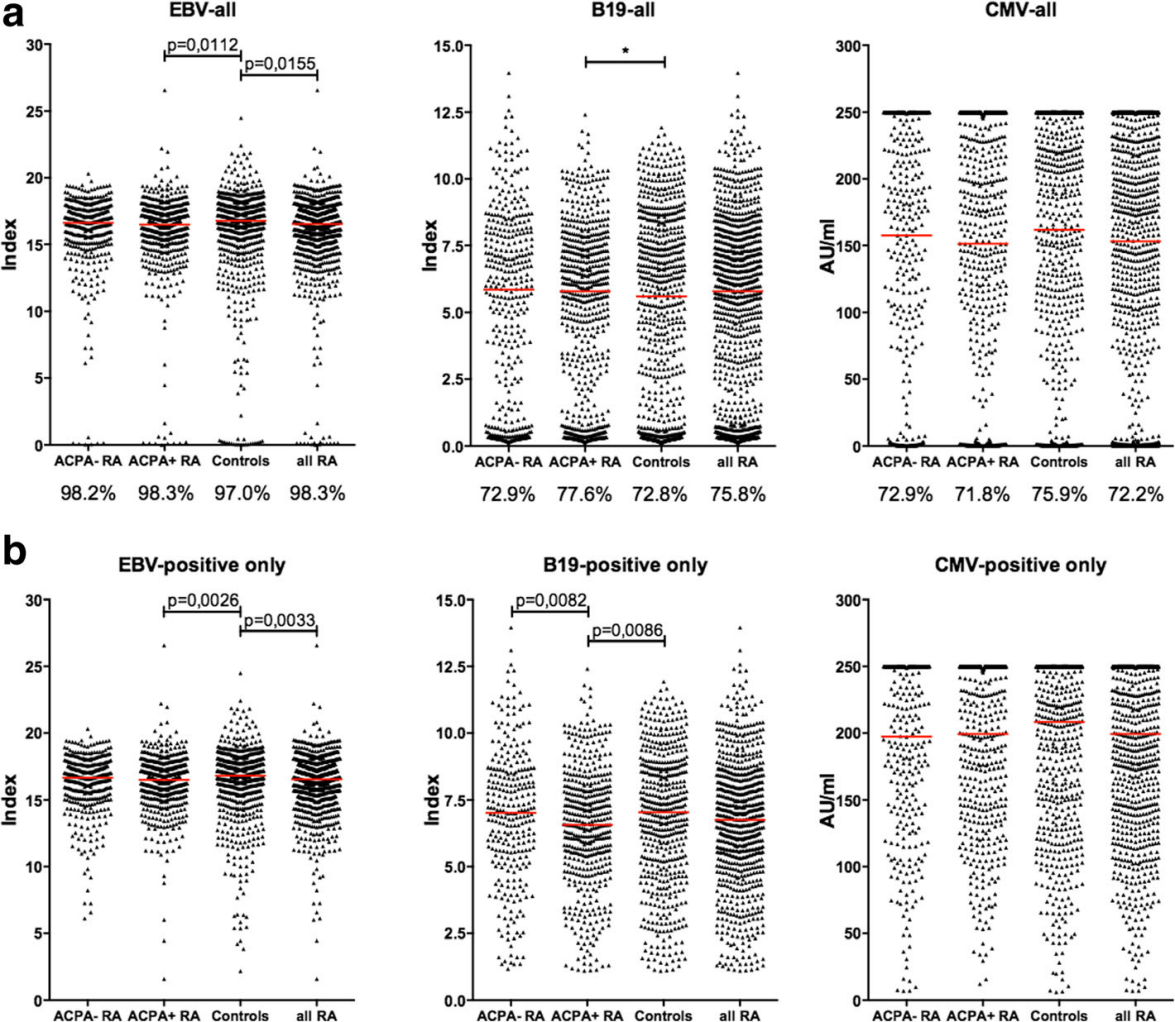
Anti-viral antibody levels in the Swedish Epidemiological Investigation of Rheumatoid Arthritis (EIRA) study: patients with rheumatoid arthritis (RA) and controls. Anti-Epstein-Barr virus (EBV) IgG (left panel), anti-B19 IgG (middle panel) and anti-cytomegalovirus (CMV) IgG (right panel) levels are shown for all study subjects (**a**) or only for anti-viral antibody-positive study subjects (**b**). The red lines indicate median antibody levels. The prevalence (%) of anti-viral antibody-positive individuals in each subset is presented below the graphs in **a**. *P* values <0.05 were considered statistically significant. *Statistical difference in antibody frequency between patients with ACPA-positive RA and controls (*p* = 0.0466) (**a**) (middle panel)

In order to determine whether infection with the common viruses CMV, EBV and parvovirus B19 was more frequent in patients with RA than in non-RA subjects, we measured anti-viral antibodies in serum from 990 patients with RA and 700 matched controls, using presence of anti-viral antibodies as surrogate markers for viral infection. We detected high frequencies of anti-viral antibody-positive individuals in the EIRA cohort, with no statistical differences between patients with RA and controls in the prevalence of any of the viruses. The prevalence of anti-EBV IgG was 98.3% and 97.0%, in patients with RA and controls, respectively; anti-B19 IgG was detected in 75.8% of patients with RA and in 72.8% of controls; and anti-CMV IgG was found in 72.2% of patients with RA and in 75.9% of controls. When dividing patients with RA into ACPA-positive and ACPA-negative subsets, there was a marginally higher frequency of anti-B19 IgG-positive individuals in ACPA-positive RA compared to controls (77.6% versus 72.8%, respectively, *p* = 0.0466) (Fig. 1a). When looking at the combination of anti-EBV, anti-B19 and anti-CMV antibodies we found no major differences between patients with RA and controls, or between patients with ACPA-positive and ACPA-negative RA. Triple positivity was most common (55–57%), followed by double positivity (34–36%), while only 8–11% were single positive, with EBV being the most common reactivity. Less than 1% was triple negative (Additional file 1: Figure S1).

### Anti-viral antibody levels in the EIRA cohort

Since we found no major differences in anti-viral antibody prevalence between groups, we proceeded to compare antibody levels. When including all individuals in these analyses, irrespective of anti-viral antibody status, only anti-EBV IgG levels differed significantly, with lower antibody levels detected in patients with RA compared to controls (*p* = 0.0155), specifically in ACPA-positive RA (*p* = 0.0112) (Fig. 1a). When focusing the analysis on anti-viral antibody-positive individuals only, both anti-EBV and anti-B19 IgG levels were significantly lower in ACPA-positive RA compared to controls (*p* = 0.0026 and *p* = 0.0086, respectively) (Fig. 1b). Anti-B19 antibody levels were also significantly lower in ACPA-positive RA compared to ACPA-negative RA (*p* = 0.0082). In order to address the possibility that RF, which is more commonly detected in ACPA-positive RA than in ACPA-negative RA, was interfering with the detection of anti-viral antibodies and thereby caused lower anti-EBV and anti-B19 IgG levels in the ACPA-positive subset, we analysed the levels of IgM RF in relation to all three anti-viral antibody levels in all EIRA RA samples. We found no correlation, hence conclude that RF did not affect anti-viral antibody levels (Additional file 2: Figure S2).

### Inverse association between high anti-viral antibody levels and RA

Due to the high frequency of seropositivity for all three anti-viral antibodies, further analyses based on seropositivity versus seronegativity would be non-informative. Instead, we analysed quartiles of anti-viral antibody levels and compared the highest quartile (25%) with the lower three quartiles (75%), in order to investigate whether high levels of anti-viral antibodies were associated with RA and/or different subsets of RA. Logistic regression analysis revealed that high levels of anti-viral antibodies did not increase the risk of RA. If anything, the opposite was observed. High levels of anti-viral antibodies were inversely associated with RA, with statistically significant odds ratios for both anti-EBV (OR = 0.7; 95% CI 0.5–0.9) and anti-B19 IgG (OR = 0.7; 95% CI 0.6–0.9). The significant associations were only confined to the ACPA-positive subset, with odds ratios of 0.6 (95% CI 0.5–0.8) for both EBV and B19 (Table 2). There was no association between ACPA-negative disease and any of the anti-viral antibodies investigated.

**Table 2:** Association between high levels of anti-viral antibodies and RA in subgroups of patients, divided based on the presence/absence of ACPA

Groups	Virus	Cases (%)	Controls (%)	OR (95% CI)^a^
All RA	EBV low^b^	805 (81)	525 (75)	1.0 ref
All RA	EBV high^c^	185 (19)	175 (25)	*0.7 (0.5–0.9)*
ACPA- RA	EBV low	307 (79)	525 (75)	1.0 ref
ACPA- RA	EBV high	82 (21)	175 (25)	0.8 (0.6–1.1)
ACPA+ RA	EBV low	498 (83)	525 (75)	1.0 ref
ACPA+ RA	EBV high	103 (17)	175 (25)	*0.6 (0.5–0.8)*
All RA	B19 low	796 (80)	525 (75)	1.0 ref
All RA	B19 high	194 (20)	175 (25)	*0.7 (0.6–0.9)*
ACPA- RA	B19 low	296 (76)	525 (75)	1.0 ref
ACPA- RA	B19 high	93 (24)	175 (25)	0.9 (0.7–1.2)
ACPA+ RA	B19 low	500 (83)	525 (75)	1.0 ref
ACPA+ RA	B19 high	101 (17)	175 (25)	*0.6 (0.5–0.8)*
All RA	CMV low	753 (76)	524 (75)	1.0 ref
All RA	CMV high	234 (24)	175 (25)	0.9 (0.7–1.2)
ACPA- RA	CMV low	296 (76)	524 (75)	1.0 ref
ACPA- RA	CMV high	91 (24)	175 (25)	1.0 (0.8–1.3)
ACPA+ RA	CMV low	457 (76)	524 (75)	1.0 ref
ACPA+ RA	CMV high	143 (24)	175 (25)	0.9 (0.7–1.2)

*RA* rheumatoid arthritis, *ACPA* anti-citrullinated protein antibody, *CI* confidence interval, *EBV* Epstein-Barr virus, *CMV* cytomegalovirus, *ref* reference

^a^Odds ratios (OR) were adjusted for age, gender and residential area. Significant ORs are shown in italics

^b^Low indicates lower three quartiles of antibody titres (75% lowest)

^c^High indicates highest quartile of antibody titres (25% highest)

In addition, we analysed different combinations of high or low anti-viral antibody levels in RA (ACPA-positive and ACPA-negative) and controls. In line with the data presented in Table 2, this analysis clearly illustrated that patients with ACPA-positive RA differed from controls, and from patients with ACPA-negative disease (Additional file 3: Figure S3). Having low antibody levels to all viruses was the most common combination (44% in controls; 46% in ACPA-negative RA; 53% in ACPA-positive RA), followed by low antibody levels to two viruses (i.e. high antibody levels to one virus - B19 in controls and ACPA-negative RA, and CMV in ACPA-positive RA).

### Anti-viral antibodies in relation to smoking and SE in ACPA-positive RA

We next analysed the associations and interactions of anti-viral antibody levels with two well-established risk factors for ACPA-positive RA, the *HLA-DRB1* SE alleles and smoking. Also here, we compared the highest quartile (25%) of antibody levels with the lower three quartiles (75%). Since high levels of anti-viral antibodies seemed to be protective against ACPA-positive RA, while presence of smoking and SE are well-known risk factors, the referent groups in these analyses were negative for SE and/or smoking, but positive for the highest quartile of anti-viral antibody levels. There was no significant association of low anti-EBV or low anti-CMV antibody levels with ACPA-positive RA in the absence of smoking (Table 3). However, low levels of anti-B19 antibodies were - independent of smoking - associated with ACPA-positive RA (OR = 2.3; 95% CI 1.3–4.1). In the presence of smoking, the odds ratio for this specific association increased to 4.4 (95% CI 2.5–7.7). No signs of interactions, calculated as the attributable proportion (AP) due to additive interaction, were identified between smoking and low levels of any of the anti-viral antibodies.

**Table 3:** Association and additive interaction between smoking and anti-viral antibodies in ACPA-positive RA

Factors		Cases (%)	Controls (%)	OR (95% CI)^a^
Smoking	EBV			
-	high^b^	20 (3.82)	55 (9.06)	1.0 ref
-	low^c^	126 (24.09)	197 (32.45)	1.6 (0.9–2.8)
+	high	73 (13.96)	96 (15.82)	*2.1 (1.1–3.9)*
+	low	304 (58.13)	259 (42.67)	*3.2 (1.9 − 5.6)*
AP (95% CI)		0.2 (-0.2–0.5)		
Smoking	B19			
-	high	18 (3.44)	61 (10.05)	1.0 ref
-	low	128 (24.47)	191 (31.47)	*2.3 (1.3–4.1)*
+	high	69 (13.19)	89 (14.66)	*2.8 (1.5–5.2)*
+	low	308 (58.89)	266 (43.82)	*4.4 (2.5–7.7)*
AP (95% CI)		0.1 (-0.3–0.4)		
Smoking	CMV			
-	high	24 (4.59)	51 (8.40)	1.0 ref.
-	low	122 (23.33)	201 (33.11)	1.4 (0.8–2.3)
+	high	109 (20.84)	100 (16.47)	*2.6 (1.5–4.6)*
+	low	268 (51.24)	255 (42.01)	*2.5 (1.5–4.3)*
AP (95% CI)		-0.2 (-0.6–0.3)		

*ACPA* anti-citrullinated protein antibody, *RA* rheumatoid arthritis, *AP* attributable proportion due to additive interaction, *CI* confidence interval, *EBV* Epstein-Barr virus, *CMV* cytomegalovirus, *ref* reference

^a^Odds ratios (OR) were adjusted for age, gender and residential area. Significant ORs are shown in italics

^b^High indicates the highest quartile of antibody titres (25% highest)

^c^Low indicates the lower three quartiles of antibody titres (75% lowest)

Analyses of *HLA-DRB1* SE in the context of anti-viral antibodies identified significant interaction between SE and low levels of both anti-EBV and anti-B19 antibodies, with an AP due to the interaction of 0.5 (95% CI 0.3–0.7) and 0.3 (95% CI 0.1–0.5), respectively (Table 4). Low levels of anti-B19 antibodies were also independently (of *HLA-DRB1* SE) associated with ACPA-positive RA. Low anti-CMV antibody levels were not associated with ACPA-positive RA and there was no interaction with SE.

**Table 4:** Association and additive interaction between *HLA-DRB1* SE alleles and anti-viral antibodies in ACPA-positive RA

Factors		Cases (%)	Controls (%)	OR (95% CI)^a^
*HLA-DRB1* SE	EBV			
-	high^b^	17 (2.83)	76 (11.00)	1.0 ref
-	low^c^	76 (12.67)	273 (39.51)	1.2 (0.6–2.1)
+	high	85 (14.17)	97 (14.03)	*3.9 (2.1–7.2)*
+	low	422 (70.33)	245 (35.46)	*7.9 (4.5–13.8)*
AP (95% CI)		*0.5 (0.3–0.7)*		
*HLA-DRB1* SE	B19			
-	high	9 (1.50)	77 (11.14)	1.0 ref
-	low	84 (14.00)	272 (39.36)	*2.7 (1.3–5.7)*
+	high	92 (15.33)	96 (13.89)	*8.8 (4.1–18.8)*
+	low	415 (69.17)	246 (35.60)	*15.7 (7.7–32.2)*
AP (95% CI)		*0.3 (0.1–0.5)*		
*HLA-DRB1* SE	CMV			
-	high	17 (2.84)	88 (12.75)	1.0 ref
-	low	76 (12.69)	261 (37.83)	1.5 (0.9–2.8)
+	high	125 (20.87)	86 (12.46)	*8.2 (4.5–15.0)*
+	low	381 (63.60)	255 (36.96)	*8.4 (4.8–14.6)*
AP (95% CI)		-0.05 (-0.4–0.3)		

*ACPA* anti-citrullinated protein antibody, *RA* rheumatoid arthritis, *AP* attributable proportion due to additive interaction, *CI* confidence interval, *EBV* Epstein-Barr virus, *CMV* cytomegalovirus, *ref* reference

^a^Odds ratios (OR) were adjusted for age, gender, and residential area. Significant ORs are shown in italics

^b^High indicates highest quartile of antibody titres (25% highest)

^c^Low indicates lower three quartiles of antibody titres (75% lowest)

**Fig. 2:**
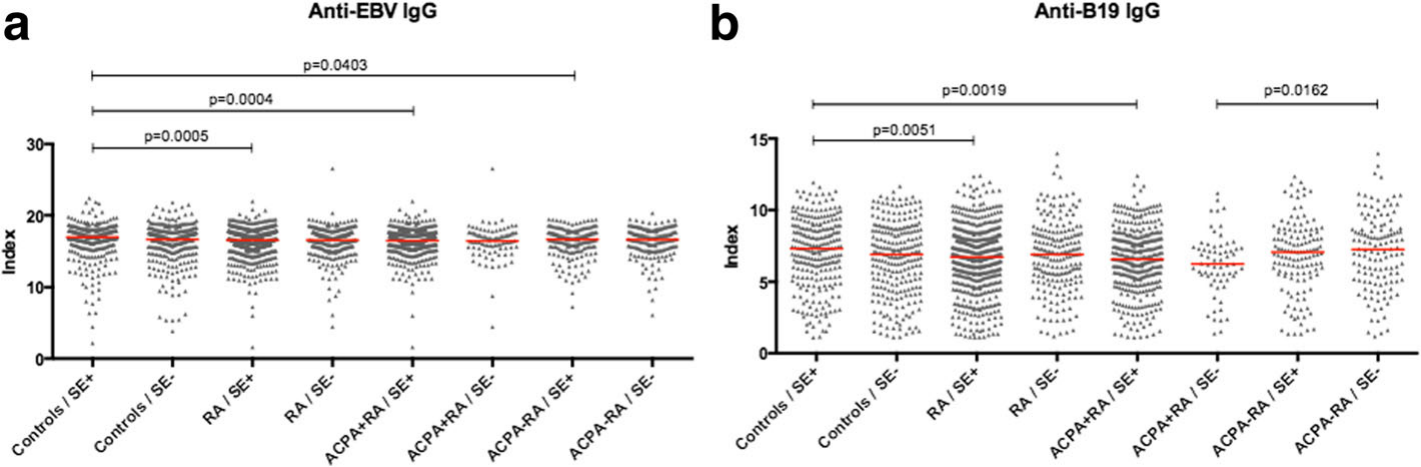
Anti-viral antibody levels in patients with rheumatoid arthritis (RA) from the Swedish Epidemiological Investigation of Rheumatoid Arthritis (EIRA) study and in controls, in relation to *HLA-DRB1* shared epitope (SE) alleles. Anti-Epstein-Barr virus (EBV) IgG (**a**) and anti-B19 IgG (**b**) levels were compared between SE-positive and SE-negative: controls, patients with RA, anti-citrullinated protein antibody (ACPA)-positive patients with RA and ACPA-negative patients with RA, and were compared in different subsets of patients with RA and in controls in the presence or absence of the SE. The red lines indicate median antibody levels. *P* values <0.05 were considered statistically significant

In order to better clarify the role of SE in relation to lower anti-viral antibody levels, as seen in ACPA-positive RA, we also compared anti-EBV and anti-B19 IgG levels in SE-positive and SE-negative subsets of patients with ACPA-positive RA, patients with ACPA-negative RA and controls. Within each subgroup, anti-viral antibody levels did not differ significantly based on SE status (Fig. 2), hence SE does not seem to directly influence these specific anti-viral antibody levels. Within the SE-positive subset, anti-B19 and anti-EBV IgG levels were significantly lower in RA and ACPA-positive RA (and also in ACPA-negative RA for EBV) compared to controls, while anti-B19 IgG levels were significantly lower in ACPA-positive RA compared to ACPA-negative RA, when comparisons were made within the SE-negative subset.

### Anti-viral antibody levels in relation to age, inflammation and disease activity

Finally, we examined whether there was correlation between anti-viral antibody levels and baseline clinical characteristics, specifically inflammation (measured as CRP) and disease activity (measured as the DAS28), and we found no correlation for any virus, in any subset of RA (Additional file 4: Figure S4). Age, on the other hand, correlated weakly with lower anti-B19 IgG levels and higher anti-CMV IgG levels, in both patients with RA and controls (Additional file 5: Figure S5).

## Discussion

In this study, we have investigated a possible association between infection by common viruses and the development of RA. To our knowledge, this is the largest epidemiological study conducted to date, where the prevalence of anti-EBV, anti-CMV and anti-B19 antibodies was investigated in a population-based RA case-control cohort. For the first time, these viruses have been examined in the context of ACPA, and genetic (*HLA-DRB1* SE) and environmental (smoking) risk factors, which today are known to contribute to disease development only in subsets of RA.

The frequencies of anti-viral antibodies were high in EIRA, and rather similar when comparing patients with ACPA-positive RA, patients with ACPA-negative RA and controls. We detected no significant differences in anti-EBV or anti-CMV IgG, while the prevalence of anti-B19 antibody-positive individuals was somewhat higher in ACPA-positive RA compared to controls. Interestingly, we found significant associations between low anti-EBV and low anti-B19 IgG levels and ACPA-positive RA. Our data are thus in agreement with a number of previous reports [12, 14, 15], yet contradictory to others [4, 5, 13]. Explanations for these discrepant results could be: (1) differences in the number of study participants, where low numbers could result in insufficient statistical power and skewed data; (2) differences in storage and processing of biological material, which may affect the antibody detection assay; (3) differences in study populations, in age, ethnicity and/or disease duration; and/or (4) differences in anti-rheumatic treatments, where immune modulatory and anti-inflammatory treatments could have negative effects on antibody titres, by altering the activity of plasma cells [31, 32].

We have used the well-characterized EIRA cohort, with 990 patients with early RA and 700 matched controls in our study, providing proper controls and high statistical power. In addition, all patients in the EIRA cohort were DMARD-naïve, and serum samples were stored at minus 80 °C until examined, minimizing potential negative effects on antibody titres. We have measured anti-viral IgG levels, and used these as surrogate markers for viral infections. Others have focused on the presence of viral DNA [7, 13, 17–19, 21, 22] or viral proteins [7, 16, 20, 24] instead, or they have used other types of assays to detect anti-viral antibodies [4, 5, 12, 14, 21].

The observation of lower anti-EBV and anti-B19 IgG levels in ACPA-positive RA could point towards an inability to mount a proper antibody response against pathogens in this subset of RA. However, generally lower antibody response to pathogens is, to our knowledge, not a specific feature of ACPA-positive RA. On the contrary, we have recently shown elevated antibody levels to the oral pathogen *Porphyromonas gingivalis* in patients with ACPA-positive RA, compared to patients with ACPA-negative RA and controls [33]. The use of corticosteroids could have a negative effect on serum IgG levels [31], and although our study only included newly diagnosed DMARD-naïve patients, we have no information on the potential use of corticosteroids before RA diagnosis. Approximately 30% of all patients in the EIRA cohort are put on prednisolone when they are diagnosed [29]. Importantly though, ACPA-positive and ACPA-negative patients are clinically very similar at this time point, and prednisolone was not more frequently administered to ACPA-positive patients compared to ACPA-negative in the EIRA cohort (personal communication, Dr Saedis Seavarsdottir). Hence, we find it unlikely that the potential use of cortisone prior to this time point would differ between ACPA-positive and ACPA-negative patients, suggesting that corticosteroids could not explain the lower anti-EBV and anti-B19 IgG levels detected in the ACPA-positive subset in our study.

Low anti-viral IgG levels could reflect low viral replication, and low viral load [34]. On the other hand, one could speculate that low anti-viral antibody levels may instead indicate an insufficient anti-viral antibody response, with poor viral control, allowing for increased viral replication, resulting in higher viral load [35]. With this interpretation of data, our observation of an interaction between low anti-EBV and low anti-B19 antibody levels and *HLA-DRB1* SE would support a number of other studies that have linked EBV and B19 to SE and RA. Balandraud and colleagues, for example, have shown that patients with RA expressing HLA-DRB1*0404 (i.e. SE positive) have higher EBV viral DNA load in peripheral blood mononuclear cells (PBMCs) than patients expressing HLA-DRB1*07 (i.e. SE negative) [35], although the difference was not statistically significant (*p* = 0.08). In another study, the frequencies of *HLA-DRB1* (*01, *04 and *07) alleles were shown to be significantly higher in individuals with symptomatic acute B19 infection than in healthy controls [36]. Moreover, Saal et al. showed that SE-positive individuals with EBV DNA detected in the synovial membrane had a higher risk of developing RA than individuals who were negative for one or both of these variables [19], and Chen et al. demonstrated synergistic effects between HLA-DRB1*04 and parvovirus B19 infection in RA susceptibility [12]. Collectively, these reports suggest that EBV and B19 may constitute environmental triggers for the development of RA in genetically predisposed individuals.

A number of studies have also suggested the possibility of autoimmunity arising as a result of molecular mimicry after EBV or B19 infection [37–39]. Anti-VP1 IgG (antibodies directed against B19 structural protein) for example, have been shown to cross-react with type II collagen, a major component in hyaline cartilage, and a target of autoantibodies in RA [40]. Moreover, antibodies to citrullinated EBV peptides have been described in RA [41–44], and Rossi et al. showed that ACPA from patients with RA have higher affinity for citrullinated autoantigens (in this case histone citrullinated peptide 1), than for citrullinated peptides derived from EBV proteins (i.e. exogenous antigens) [45], suggesting that these antibodies are initially produced against exogenous antigen and later selected and expanded by autoantigens.

Recent studies have also shown that EBV can persist in self-reactive memory B cells, thereby favouring the survival of pathogenic autoreactive B cells [46, 47]. In addition, macrophages and lymphocytes infected by B19 provide another possible mechanism by which viruses could contribute to autoimmunity, through the continuous secretion of pro-inflammatory cytokines resulting in polyclonal B cell activation and proliferation of synovial B cells [7, 48–50].

A weakness of our study is that we have only analysed anti-viral IgG levels, which most probably reflect infections occurring in childhood. We have not analysed anti-viral IgM or presence of viral DNA, which could have given us information about acute infections/re-activation of the viruses. Moreover, whether low anti-viral antibody levels reflect low viral load, or the opposite, high viral load, is not clear, and may differ from virus to virus. In order to dissect the role of common viruses in RA aetiology in detail, and the relationship between viral load and the anti-viral immune response, in the context of SE and ACPA, longitudinal studies should be performed, in which viral DNA and anti-viral IgM is measured in addition to anti-viral IgG.

## Conclusion

In conclusion, our study demonstrates an inverse/negative association between high anti-EBV and high anti-B19 IgG levels and the risk of developing ACPA-positive RA. Moreover, we detected significant associations between low anti-B19 and low anti-EBV IgG levels and ACPA-positive RA, especially in the context of *HLA-DRB1* SE. Hence, our data imply that a strong immune response against parvovirus and EBV virus (which historically could have been implicated in arthritis development) may in fact be protective in a pathogenically well-defined subset of patients with RA.

## Additional files

Additional file 1: Figure S1. Prevalence of being positive for different combinations of anti-viral antibodies in RA, ACPA-positive RA, ACPA-negative RA and in controls. Seropositivity for one, two, three or no anti-viral antibodies are shown in **A**. Seropositivity for different combinations of anti-EBV, anti-B19 and anti-CMV IgG are shown in **B**. (EPS 111 kb)

Additional file 2: Figure S2. Correlation between anti-EBV (left panel), anti-B19 (middle panel) and anti-CMV (right panel) IgG levels and IgM rheumatoid factor (RF) levels in all patients with RA (upper row), ACPA-positive patients with RA (middle row) and ACPA-negative patients with RA (lower row). (EPS 1873 kb)

Additional file 3: Figure S3. Prevalence of having different combinations of high and low anti-EBV, anti-B19 and anti-CMV antibody levels in RA, ACPA-positive RA, ACPA-negative RA, and in controls. “High” indicates the highest quartile of antibody titres (25% highest). “Low” indicates the lower three quartiles of antibody titres (75% lowest). (EPS 89 kb)

Additional file 4: Figure S4. Correlations between anti-EBV (left panel), anti-B19 (middle panel), anti-CMV (right panel) IgG levels and C-reactive protein (CPR) levels (**A**) and disease activity score (DAS28) (**B**) in all patients with RA (upper row), in ACPA-positive patients with RA (middle row) and in ACPA-negative patients with RA (lower row). (EPS 1848 kb)

Additional file 5: Figure S5. Correlations between anti-EBV (left panel), anti-B19 (middle panel), anti-CMV (right panel) IgG levels and age in patients with RA (**A**) and in controls (**B**). (EPS 1320 kb)

## Abbreviations

ACPA: Anti-citrullinated protein antibody
AP: Attributable proportion
AU: Arbitrary units
B19: Human parvovirus B19
CI: Confidence intervals
CMV: Cytomegalovirus
CRP: C-reactive protein
DAS28: Disease activity score in 28 joints
DMARD: Disease-modifying drug
EBV: Epstein-Barr virus
EIRA: Swedish Epidemiological Investigation of Rheumatoid Arthritis
ELISA: Enzyme-linked immunosorbent assay
IQR: Interquartile range
N/A: Not applicable
OR: Odds ratio
PBMC: Peripheral blood mononuclear cells
RA: Rheumatoid arthritis
RF: Rheumatoid factor
SD: Standard deviation
SE: Shared epitope

## References

[1] Gregersen PK, Silver J, Winchester RJ (1987). The shared epitope hypothesis. An approach to understanding the molecular genetics of susceptibility to rheumatoid arthritis. Arthritis Rheum.

[2] Stolt P, Bengtsson C, Nordmark B, Lindblad S, Lundberg I, Klareskog L (2003). Quantification of the influence of cigarette smoking on rheumatoid arthritis: results from a population based case-control study, using incident cases. Ann Rheum Dis.

[3] Klareskog L, Stolt P, Lundberg K, Källberg H, Bengtsson C, Grunewald J (2006). A new model for an etiology of rheumatoid arthritis: smoking may trigger HLA-DR (shared epitope)-restricted immune reactions to autoantigens modified by citrullination. Arthritis Rheum.

[4] Alspaugh MA, Henle G, Lennette ET, Henle W (1981). Elevated levels of antibodies to Epstein-Barr virus antigens in sera and synovial fluids of patients with rheumatoid arthritis. J Clin Invest.

[5] Ferrell PB, Aitcheson CT, Pearson GR, Tan EM (1981). Seroepidemiological study of relationships between Epstein-Barr virus and rheumatoid arthritis. J Clin Invest.

[6] Costenbader KH, Karlson EW (2006). Epstein-Barr virus and rheumatoid arthritis: is there a link?. Arthritis Res Ther.

[7] Takahashi Y, Murai C, Shibata S, Munakata Y, Ishii T, Ishii K (1998). Human parvovirus B19 as a causative agent for rheumatoid arthritis. Proc Natl Acad Sci U S A.

[8] Kerr JR (2000). Pathogenesis of human parvovirus B19 in rheumatic disease. Ann Rheum Dis.

[9] Soderberg-Naucler C (2006). Does cytomegalovirus play a causative role in the development of various inflammatory diseases and cancer?. J Intern Med.

[10] White DG, Woolf AD, Mortimer PP, Cohen BJ, Blake DR, Bacon PA (1985). Human parvovirus arthropathy. Lancet.

[11] Woolf AD, Campion GV, Chishick A, Wise S, Cohen BJ, Klouda PT (1989). Clinical manifestations of human parvovirus B19 in adults. Arch Intern Med.

[12] Chen YS, Chou PH, Li SN, Tsai WC, Lin KH, Tsai KB (2006). Parvovirus B19 infection in patients with rheumatoid arthritis in Taiwan. J Rheumatol.

[13] Saal JG, Steidle M, Einsele H, Müller CA, Fritz P, Zacher J (1992). Persistence of B19 parvovirus in synovial membranes of patients with rheumatoid arthritis. Rheumatol Int.

[14] Goldstein BL, Chibnik LB, Karlson EW, Costenbader KH (2012). Epstein-Barr virus serologic abnormalities and risk of rheumatoid arthritis among women. Autoimmunity.

[15] Pierer M, Rothe K, Quandt D, Schulz A, Rossol M, Scholz R (2012). Association of anticytomegalovirus seropositivity with more severe joint destruction and more frequent joint surgery in rheumatoid arthritis. Arthritis Rheum.

[16] Mehraein Y, Lennerz C, Ehlhardt S, Venzke T, Ojak A, Remberger K (2003). Detection of parvovirus B19 capsid proteins in lymphocytic cells in synovial tissue of autoimmune chronic arthritis. Mod Pathol.

[17] Mehraein Y, Lennerz C, Ehlhardt S, Remberger K, Ojak A, Zang KD (2004). Latent Epstein-Barr virus (EBV) infection and cytomegalovirus (CMV) infection in synovial tissue of autoimmune chronic arthritis determined by RNA- and DNA-in situ hybridization. Mod Pathol.

[18] Stahl HD, Hubner B, Seidl B, Liebert UG, van der Heijden IM, Wilbrink B (2000). Detection of multiple viral DNA species in synovial tissue and fluid of patients with early arthritis. Ann Rheum Dis.

[19] Saal JG, Krimmel M, Steidle M, Liebert UG, van der Heijden IM (1999). Synovial Epstein-Barr virus infection increases the risk of rheumatoid arthritis in individuals with the shared HLA-DR4 epitope. Arthritis Rheum.

[20] Takei M, Mitamura K, Fujiwara S, Horie T, Ryu J, Osaka S (1997). Detection of Epstein-Barr virus-encoded small RNA 1 and latent membrane protein 1 in synovial lining cells from rheumatoid arthritis patients. Int Immunol.

[21] Murai C, Munakata Y, Takahashi Y, Ishii T, Shibata S, Muryoi T (1999). Rheumatoid arthritis after human parvovirus B19 infection. Ann Rheum Dis.

[22] Dijkmans BA, van Elsacker-Niele AM, Salimans MM, van Albada-Kuipers GA, de Vries E, Weiland HT (1988). Human parvovirus B19 DNA in synovial fluid. Arthritis Rheum.

[23] Lundqvist A, Isa A, Tolfvenstam T, Kvist G, Broliden K (2005). High frequency of parvovirus B19 DNA in bone marrow samples from rheumatic patients. J Clin Virol.

[24] Niedobitek G, Lisner R, Swoboda B, Rooney N, Fassbender HG, Kirchner T (2000). Lack of evidence for an involvement of Epstein-Barr virus infection of synovial membranes in the pathogenesis of rheumatoid arthritis. Arthritis Rheum.

[25] Peterlana D, Puccetti A, Beri R, Ricci M, Simeoni S, Borgato L (2003). The presence of parvovirus B19 VP and NS1 genes in the synovium is not correlated with rheumatoid arthritis. J Rheumatol.

[26] Nikkari S, Roivainen A, Hannonen P, Möttönen T, Luukkainen R, Yli-Jama T (1995). Persistence of parvovirus B19 in synovial fluid and bone marrow. Ann Rheum Dis.

[27] Arnett FC, Edworthy SM, Bloch DA, McShane DJ, Fries JF, Cooper NS (1988). The American Rheumatism Association 1987 revised criteria for the classification of rheumatoid arthritis. Arthritis Rheum.

[28] Padyukov L, Silva C, Stolt P, Alfredsson L, Klareskog L (2004). A gene-environment interaction between smoking and shared epitope genes in HLA-DR provides a high risk of seropositive rheumatoid arthritis. Arthritis Rheum.

[29] Saevarsdottir S, Wedrén S, Seddighzadeh M, Bengtsson C, Wesley A, Lindblad S (2011). Patients with early rheumatoid arthritis who smoke are less likely to respond to treatment with methotrexate and tumor necrosis factor inhibitors: observations from the Epidemiological Investigation of Rheumatoid Arthritis and the Swedish Rheumatology Register cohorts. Arthritis Rheum.

[30] Hosmer D, Lemeshow S (1992). Confidence interval estimation of interaction. Epidemiology.

[31] Settipane GA, Pudupakkam RK, McGowan JH (1978). Corticosteroid effect on immunoglobulins. J Allergy Clin Immunol.

[32] Fedor ME, Rubinstein A (2006). Effects of long-term low-dose corticosteroid therapy on humoral immunity. Ann Allergy Asthma Immunol.

[33] Kharlamova N, Jiang X, Sherina N, Potempa B, Israelsson L, Quirke AM (2016). Antibodies to Porphyromonas gingivalis indicate interaction between oral infection, smoking, and risk genes in rheumatoid arthritis etiology. Arthritis Rheumatol.

[34] Besson C, Amiel C, Le-Pendeven C, Brice P, Fermé C, Carde P (2006). Positive correlation between Epstein-Barr virus viral load and anti-viral capsid immunoglobulin G titers determined for Hodgkin's lymphoma patients and their relatives. J Clin Microbiol.

[35] Balandraud N, Meynard JB, Auger I, Sovran H, Mugnier B, Reviron D (2003). Epstein–Barr virus load in the peripheral blood of patients with rheumatoid arthritis, accurate quantification using real time polymerase chain reaction. Arthritis Rheum.

[36] Kerr JR, Mattey DL, Thomson W, Poulton KV, Ollier WE (2002). Association of symptomatic acute human parvovirus B19 infection with human leukocyte antigen class I and II alleles. J Infect Dis.

[37] Münz C, Lünemann JD, Getts MT, Miller SD (2009). Antiviral immune responses: triggers of or triggered by autoimmunity?. Nat Rev Immunol.

[38] Pavlovic M, Kats A, Cavallo M, Shoenfeld Y. Clinical and molecular evidence for association of SLE with parvovirus B19. Lupus. 2010;19(7):783–92. PMID: 20511275. 10.1177/0961203310365715 20511275

[39] Lunardi C, Tinazzi E, Bason C, Dolcino M, Corrocher R, Puccetti A (2008). Human parvovirus B19 infection and autoimmunity. Autoimmun Rev.

[40] Lunardi C, Tiso M, Borgato L, Nanni L, Millo R, De Sandre G (1998). Chronic parvovirus B19 infection induces the production of anti-virus antibodies with autoantigen binding properties. Eur J Immunol.

[41] Cornillet M, Verrouil E, Cantagrel A, Serre G, Nogueira L (2015). In ACPA-positive RA patients, antibodies to EBNA35-58Cit, a citrullinated peptide from the Epstein-Barr nuclear antigen-1, strongly cross-react with the peptide β60-74Cit which bears the immunodominant epitope of citrullinated fibrin. Immunol Res.

[42] Anzilotti C, Merlini G, Pratesi F, Tommasi C, Chimenti D, Migliorini P (2006). Antibodies to viral citrullinated peptide in rheumatoid arthritis. J Rheumatol.

[43] Pratesi F, Tommasi C, Anzilotti C, Chimenti D, Migliorini P (2006). Deiminated Epstein-Barr virus nuclear antigen 1 is a target of anti-citrullinated protein antibodies in rheumatoid arthritis. Arthritis Rheum.

[44] Pratesi F, Tommasi C, Anzilotti C, Puxeddu I, Sardano E, Di Colo G (2011). Antibodies to a new viral citrullinated peptide, VCP2: fine specificity and correlation with anti-cyclic citrullinated peptide (CCP) and anti-VCP1 antibodies. Clin Exp Immunol.

[45] Rossi G, Real-Fernández F, Panza F, Barbetti F, Pratesi F, Rovero P (2014). Biosensor analysis of anti-citrullinated protein/peptide antibody affinity. Anal Biochem.

[46] Croia C, Serafini B, Bombardieri M, Kelly S, Humby F, Severa M (2013). Epstein-Barr virus persistence and infection of autoreactive plasma cells in synovial lymphoid structures in rheumatoid arthritis. Ann Rheum Dis.

[47] Brisslert M, Rehnberg M, Bokarewa MI (2013). Epstein-Barr virus infection transforms CD25+ B cells into antibody-secreting cells in rheumatoid arthritis patients. Immunology.

[48] Moffatt S, Tanaka N, Tada K, Nose M, Nakamura M, Muraoka O (1996). A cytotoxic nonstructural protein, NS1, of human parvovirus B19 induces activation of interleukin-6 gene expression. J Virol.

[49] Tzang BS, Chiu CC, Tsai CC, Lee YJ, Lu IJ, Shi JY (2009). Effects of human parvovirus B19 VP1 unique region protein on macrophage responses. J Biomed Sci.

[50] Kerr JR, Barah F, Mattey DL, Laing I, Hopkins SJ, Hutchinson IV (2001). Circulating tumour necrosis factor-alpha and interferon-gamma are detectable during acute and convalescent parvovirus B19 infection and are associated with prolonged and chronic fatigue. J Gen Virol.

